# Influence of Temperature on Intra- and Interspecific Resource Utilization within a Community of Lepidopteran Maize Stemborers

**DOI:** 10.1371/journal.pone.0148735

**Published:** 2016-02-09

**Authors:** Eric Siaw Ntiri, Paul-Andre Calatayud, Johnnie Van Den Berg, Fritz Schulthess, Bruno Pierre Le Ru

**Affiliations:** 1 International Centre of Insect Physiology and Ecology, Nairobi, Kenya; 2 Unit of Environmental Sciences and Management, North-West University, Potchefstroom, South Africa; 3 UMR IRD 247 Laboratoire Evolution, Génomes, Comportement et Ecologie, Diversité, Ecologie et Evolution des Insectes Tropicaux, CNRS, Gif-sur-Yvette, France and Université de Paris-Sud, Orsay, France; 4 Postfach 508, Chur, Switzerland; Pennsylvania State University, UNITED STATES

## Abstract

Competition or facilitation characterises intra- and interspecific interactions within communities of species that utilize the same resources. Temperature is an important factor influencing those interactions and eventual outcomes. The noctuid stemborers, *Busseola fusca* and *Sesamia calamistis* and the crambid *Chilo partellus* attack maize in sub-Saharan Africa. They often occur as a community of interacting species in the same field and plant at all elevations. The influence of temperature on the intra- and interspecific interactions among larvae of these species, was studied using potted maize plants exposed to varying temperatures in a greenhouse and artificial stems kept at different constant temperatures (15°C, 20°C, 25°C and 30°C) in an incubator. The experiments involved single- and multi-species infestation treatments. Survival and relative growth rates of each species were assessed. Both intra- and interspecific competitions were observed among all three species. Interspecific competition was stronger between the noctuids and the crambid than between the two noctuids. Temperature affected both survival and relative growth rates of the three species. Particularly at high temperatures, *C*. *partellus* was superior in interspecific interactions shown by higher larval survival and relative growth rates. In contrast, low temperatures favoured survival of *B*. *fusca* and *S*. *calamistis* but affected the relative growth rates of all three species. Survival and relative growth rates of *B*. *fusca* and *S*. *calamistis* in interspecific interactions did not differ significantly across temperatures. Temperature increase caused by future climate change is likely to confer an advantage on *C*. *partellus* over the noctuids in the utilization of resources (crops).

## Introduction

Maize (*Zea mays* L.) is one of the most important cereal crops worldwide, utilized as human food, animal feed and industrial raw materials [1, 2]. In developed countries such as in the US (the largest producer of maize), a greater part of total production is used for animal feed, with an increased proportion utilized for biofuel [1, 2]. However, in developing countries such as in Africa, 95% of total maize production, mostly by small scale farms, is used for human food. In addition, maize production in this region is fraught with a myriad of challenges including pests, diseases, drought and nutrient deficiency [1].

Lepidopteran stemborers such as the indigenous noctuids *Busseola fusca* (Fuller) and *Sesamia calamistis* (Hampson) and the exotic crambid *Chilo partellus* (Swinhoe) attack the maize crop in East and southern Africa [3, 4]. Depending on the elevation they may occur as single species or communities of mixed species attacking maize stems in the same field [5–8]. For instance, in Kenya, the composition of these stemborer communities varies with elevation. *Busseola fusca* is the predominant species in the highlands characterised by low temperatures, while *C*. *partellus* is the most abundant species in the hot lowlands. In contrast, *S*. *calamistis* is present in low numbers at all elevations. It is only at the mid-elevations do the three species occur as a mixed community, but the predominance of a species may vary with location and season [7, 9, 10].

The common use of a limited resource by several species for their survival predisposes them to interact competitively [11–13] or facilitatively [11, 14, 15]. Some of the most important questions in ecology concern intra- and interspecies interactions in mixed communities [16]. For instance, the role of competition in the organisation of insect communities despite being questioned by several authors [11, 17–19], has been resuscitated by two crucial reviews on the subject which presented strong evidence for the dominance of competition in phytophagous insect communities [11, 20].

Temperature is the most crucial abiotic factor for insects, as it directly drives their life processes [21–26]. It also influences resource utilisation, intra- and inter-specific interactions and limits their geographic distribution [24, 27–32]. For example, competitive interactions between the burying beetles *Nicrophorus orbicollis* Say and *N*. *defodiens* Mannerheim (Coleoptera: Silphidae), when feeding on the same carcass, was reported to be temperature dependent [33]. In an experiment involving the seed beetle *Stator limbatus* (Horn) (Coleoptera: Chrysomelidae), cooler temperatures conferred a competitive advantage on smaller males, which out-competed larger ones in reaching a potential mate [34]. Future temperature increase due to climate change [35] is predicted to affect the type and intensity of species interactions [28, 36–38]. For example, changes in temperature was reported to influence the intensity of intraspecific competition by the grasshopper *Camnula pellucida* (Scudder) (Orthoptera: Acrididae) [39]. Surprisingly few studies have been carried out to assess the effect of possible future temperature increases on the competitive and facilitative interactions within communities of insects utilising the same resource [38, 40].

Reports of competitive displacement of *B*. *fusca* and *Chilo orichalcociliellus* Strand by *C*. *partellus* from overlap in resource use have been reported in South Africa [41, 42] and in the coastal region of Kenya [43], respectively, but the mechanisms behind the species displacements are not known. The temperature requirements of each of these stemborers have been well studied [25, 26], but the effects of temperature on their interactions are yet to be elucidated.

This paper reports on the kind of intra- and interspecific interactions that characterise resource utilization (maize infestation) by communities of *B*. *fusca*, *S*. *calamistis* and *C*. *partellus* and the effect of temperature on these interactions, as well as discusses the potential impact of climate changes on these interactions.

## Materials and Methods

### Plants and insects

Seeds of the H513 hybrid maize variety (Simlaw, Kenya Seed Company, Nairobi, Kenya) were planted in plastic pots (12 cm in height x 13 cm in diameter), in a greenhouse at the campus of the International Centre of Insect Physiology and Ecology (*icipe*), Nairobi, Kenya (S 01°13'17.8", E 036°53'45.0"). Mean temperatures were approximately 31/17°C (day/night) with a 12:12 h (L:D) photoperiod. Maize plants at the sixth leaf stage, (the earliest maize plant stage found to be infested in the field) were used in the experiments.

Second instar larvae of *B*. *fusca* (Bf), *C*. *partellus* (Cp) and *S*. *calamistis* (Sc) were obtained from colonies reared at the Animal Rearing and Containment Unit (ARCU) at *icipe*, Nairobi, Kenya. Larvae were reared in plastic jars (16.5 cm length x 9cm diameter) filled with 200 ml of artificial diet of Onyango and Ochieng’-Odero [44]. The diet contained vitamins, maize leaf powder, Brewer’s yeast, bean powder and anti-microbial agents such as ascorbic acid. Agar was also added to enable liquid diet to solidify and also to hold moisture. The jars were covered with tissue paper and tightly closed with perforated lids with galvanized mesh, to prevent larvae escape and kept in a holding room with a temperature of 26±1°C and RH of 60±5%. Colonies were rejuvenated twice a year with field collected larvae.

### Surrogate stems

**Fig 1:**
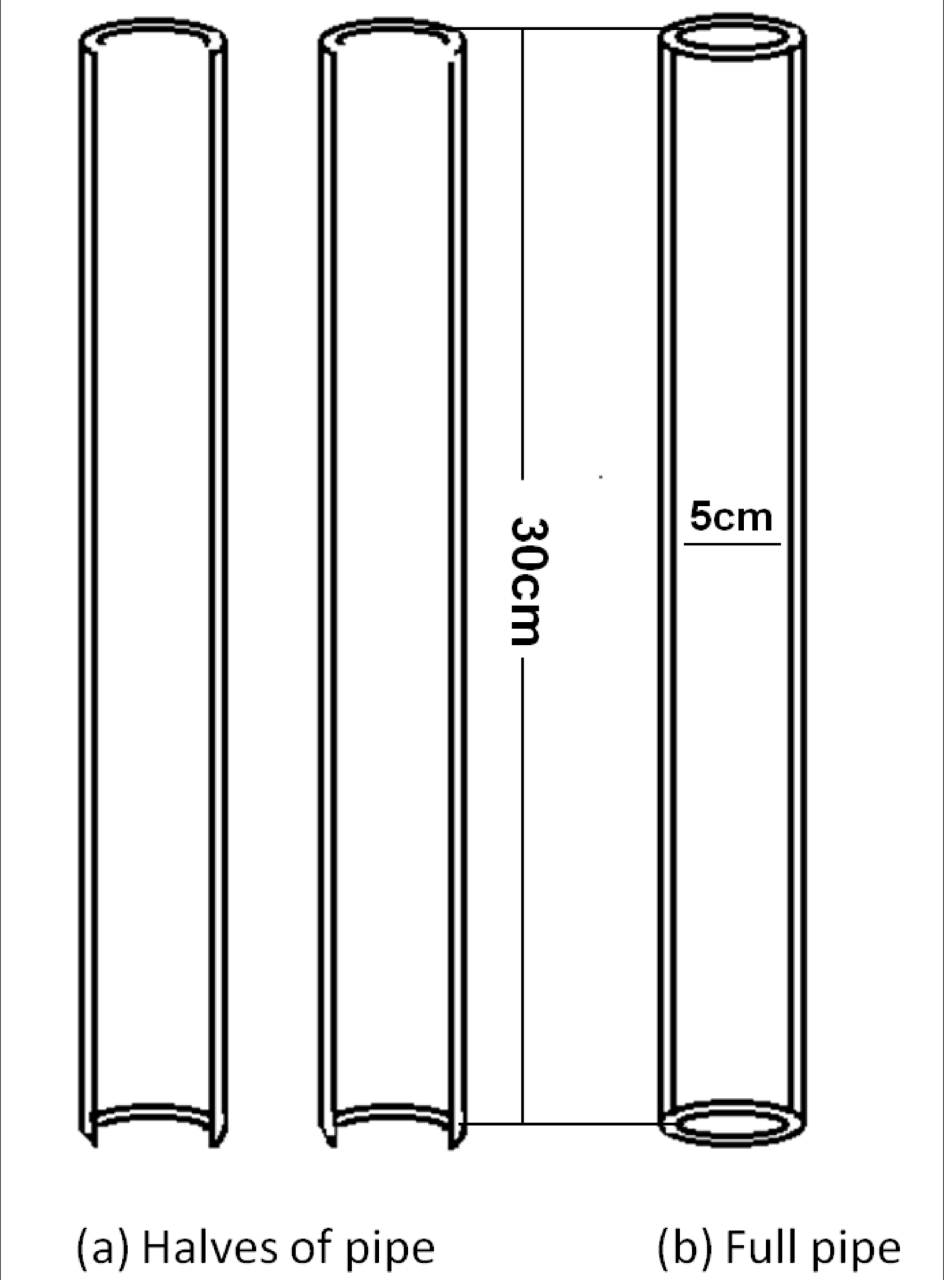
PVC surrogate stem for rearing stemborer larvae on artificial diet. (a) halves of pipe before they are joined, (b) full pipe after halves have been joined.

In a preliminary trial, larvae-infested plants kept in an incubator (Sanyo MIR 554, Japan) deteriorated after only 5–7 days. Thus, a method using surrogate stems filled with artificial diet was used (Fig 1). These surrogate stems consisted of a 30cm piece of PVC pipe with a diameter of 5cm. Each piece was cut into equal halves to allow opening of the stem for observation of the larvae. The halves were held together with masking tape. One end of the pipe was covered with parafilm® and reinforced with masking tape. The pipe was wrapped in aluminium foil and fastened with a rubber band leaving one end uncovered. This was done to prevent leakage of hot liquid diet when dispensed later into pipes. The pipes were then filled with the aforementioned artificial diet of Onyango and Ochieng’-Odero [44]. Once the diet had solidified in the tubes after 24 hours, the masking tapes and three quarters of the aluminium foil covering the tubes were removed from top to bottom, leaving only one quarter of the pipe covered.

The following three experiments were conducted:

### Experiment 1. The influence of maize and surrogate stems on the development of stemborer larvae

This experiment involved a single-species infestation treatment, conducted on potted plants and surrogate stems. Both substrates were each infested with 12 second instar larvae of the same species of each species (Cp, Bf, and Sc), using a small camel hair brush (size 2). A density of 12 larvae per surrogate stem is representative of that found on maize in the field at the beginning of the infestation (B. Le Ru, unpublished). The infested plants were covered with a netted metal frame tied with rubber bands at the base of the pots to prevent larvae from escaping. For the surrogate stems, the free ends were plugged with cotton wool after infestation with larvae. Each treatment was replicated twenty times on both maize plants and surrogate stems. The surrogate stems were placed in jars to keep them upright. The experiment was undertaken under varying temperatures in a semi-natural condition in a greenhouse during the hot season, from December to March (min. temp., 13°C, max. temp., 27°C, and mean of 20°C). This period corresponded to the growing season of maize in most parts of Kenya. The temperature was recorded with a HOBO^®^ Temp/RH data logger (Onset, USA). After 30 days, all maize stems were dissected and surrogates stems opened to record the number and the mass of surviving larvae of each species.

### Experiment 2. Influence of larval density on intra-specific interactions

This experiment was conducted to investigate intraspecific interactions at low and high density infestations. For the low density infestation, surrogate stems were infested with six second instar larvae (6L) and for the high density infestation, stems were infested with twelve second instar larvae (12L) of the same species of each species (Cp, Bf, and Sc). The surrogate stems were then plugged with cotton wool after infestation. The surrogate stems were placed in jars to keep them upright and were then kept in an incubator (Sanyo MIR 554, Japan) at a constant temperature of 25°C, the optimum temperature for all three species [25, 26], air humidity of 70±10% and LD of 12:12. Each treatment was replicated twenty times. The number and mass of surviving larvae of each species were recorded from surrogate stems after 30 days.

### Experiment 3. Influence of different constant temperatures on intra- and interspecific interactions

This experiment involved single- and multi-species infestation treatments conducted with surrogate stems. The single-species infestation treatments involved infestation of surrogate stems with 12 larvae of the same species of each species (Cp, Bf and Sc). The multi-species infestation treatment involved infestation of surrogate stems with six larvae of each species for the Cp+Bf, Cp+Sc, Bf+Sc pairings, and four larvae of each species for the three-species treatment, Cp+Bf+Sc. The surrogate stems were then plugged with cotton wool after infestation. The stems were placed in jars to keep them upright. This experiment was conducted in incubators (Sanyo MIR 554, Japan) at four constant temperatures of 15, 20, 25, and 30°C, air humidity of 70±10% and LD of 12:12. Each treatment was replicated twenty times. After 30 days, surrogates stems were opened to record the number and the mass of surviving larvae of each species.

### Data analysis

Survival rates (i.e., the number of larvae alive after 30 days) and relative growth rates (RGR) were used as the response variables. The RGR for each species was calculated following the equation of Ojeda-Avila et al.[45]:

RGR=(Total mass of surviving larvae−initial mass of larvae)/No.of days.

RGR for communities was calculated as the sum of the RGR of all species in that community. Survival rates for each treatment were analysed using the generalized linear model with binomial error structure. Odd Ratios with a 95% confidence interval (O.R. [95%CI]) were calculated for the comparison made between treatments from the GLM results obtained. The differences between RGR of species from each treatment, was analysed via analysis of variance (ANOVA). The level of significance was set at 5%. Means were separated with the Student-Newman-Keuls (SNK) test. The RGR data were first tested for normality of their distribution by a Kolmogorov-Smirnov test and for homoscedasticity by the Bartlett’s test. All analyses were carried out in R [46].

## Results

### Experiment 1. Influence of maize and surrogate stems on the development of stem borer larvae

**Fig 2:**
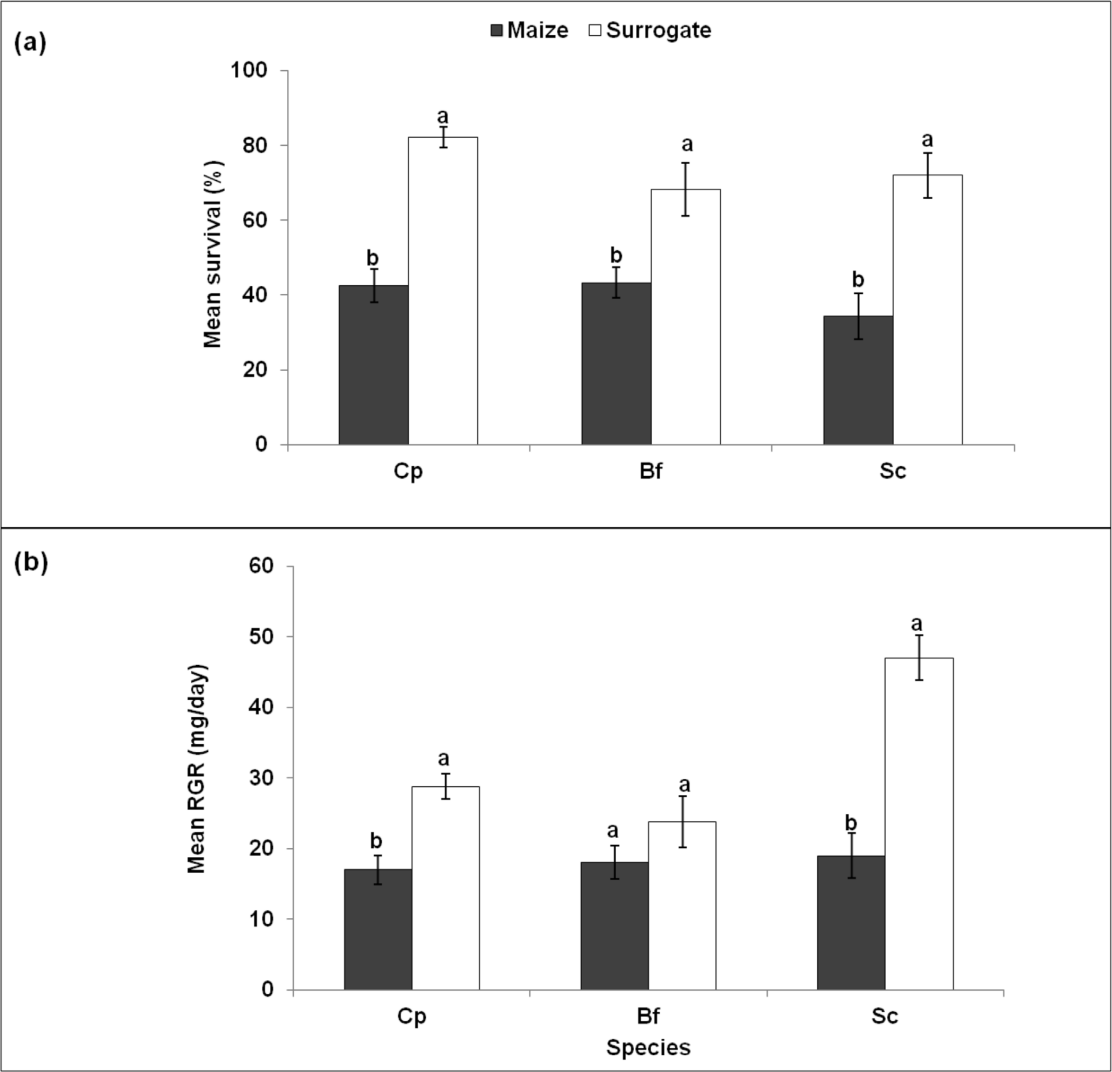
**Survival (a) and relative growth rates (b) of *Chilo partellus* (Cp), *Busseola fusca* (Bf) and *Sesamia calamistis* (Sc) larvae on maize and surrogate stems under varying temperatures.** Means (± SE) with different letters are significantly different at 5% level according to the GLM for survival and the Student-Newman-Keuls test for relative growth rates.

For each species, survival rates were significantly higher on surrogate stems than maize plants (Fig 2A). The survival of each species was about double in surrogate stems compared to maize plants. RGRs were also significantly higher on surrogate stems than on maize plants for *C*. *partellus* and *S calamistis*. It increased by a factor of 1.5 to 2 for each species respectively on surrogate stems compared to maize plants (Fig 2B). However, the RGR of *B*. *fusca* did not differ significantly between surrogate stems and maize plants.

### Experiment 2. Influence of larval density on intra-specific interactions between stem borer larvae

**Fig 3:**
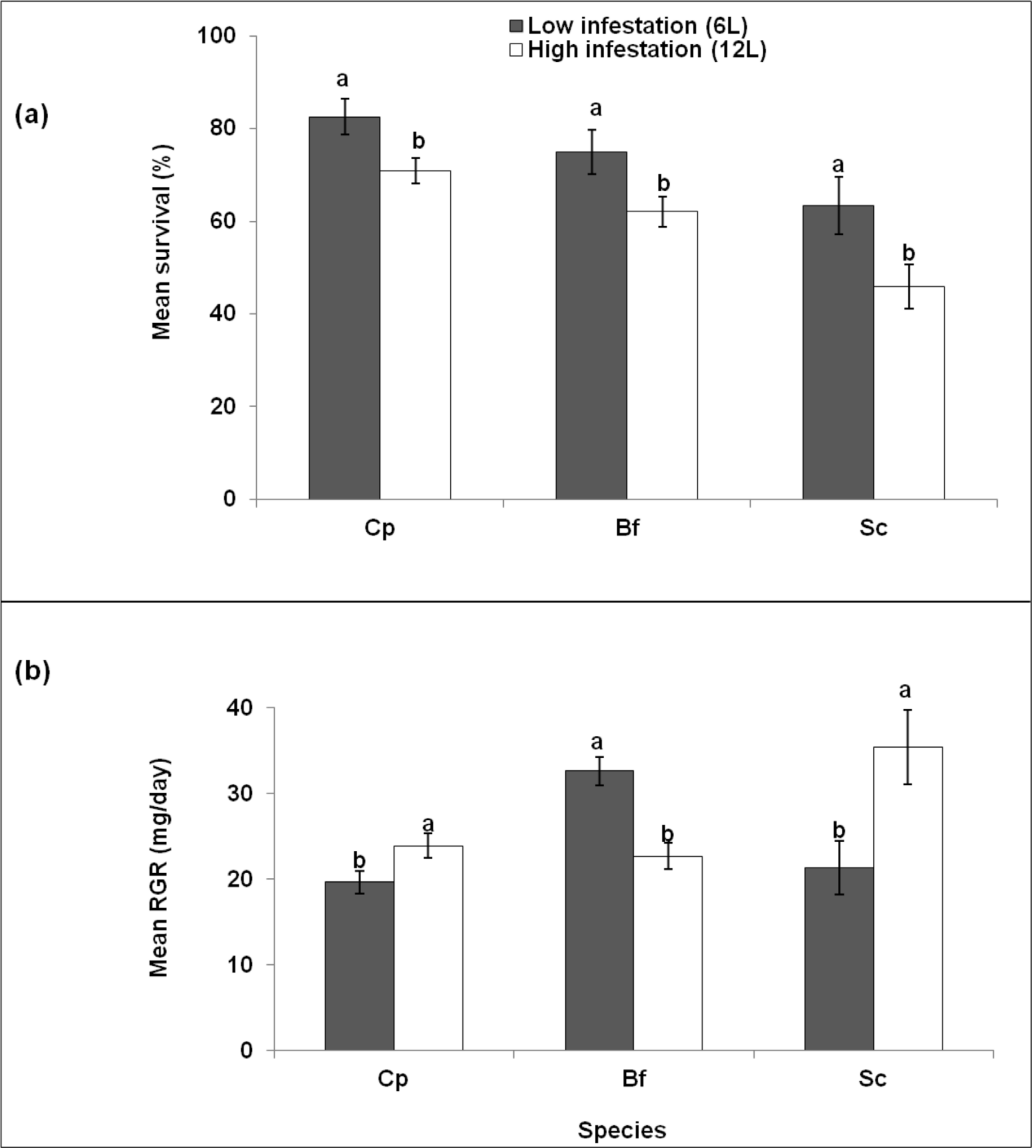
**Survival (a) and relative growth rates (b) of *Chilo partellus* (Cp), *Busseola fusca* (Bf) and *Sesamia calamistis* (Sc) at low density (6L) and high density (12L) infestation at 25°C.** Means (± SE) with different letters are significantly different at 5% level according to the GLM for survival and the Student-Newman-Keuls test for relative growth rates.

The survival rates were significantly lower for high infestation than low infestation levels for *B*. *fusca* [O.R. = 1.8 (1.06–3.21), p = 0.03], *C*. *partellus* [O.R. = 1.9 (1.1–3.47), p = 0.02] and *S*. *calamistis* [O.R. = 2.0 (1.1–3.9), p = 0.03] (Fig 3A). For both infestation levels, *C*. *partellus* had the highest survival rate, while *S*. *calamistis* had the lowest survival rates. However, there were variations in the RGRs of the three species at the different densities. The RGRs were significantly higher for high infestation than low infestation for *C*. *partellus* (F = 4.9, p = 0.03) and *S*. *calamistis* (F = 6.9, p = 0.01), whereas for *B*. *fusca* it was significantly higher (F = 19.3, p<0.001) for low infestation than high infestation levels (Fig 3B). Also, while *B*. *fusca* had the highest RGR at low infestation level, *S*. *calamistis* had the highest RGR at high infestation level (Fig 3B).

### Experiment 3. Influence of different constant temperatures on intra- and interspecific interactions

#### a) The effect of temperature on survival and RGR of *B*. *fusca*, *C*. *partellus* and *S*. *calamistis* as single-species

**Fig 4:**
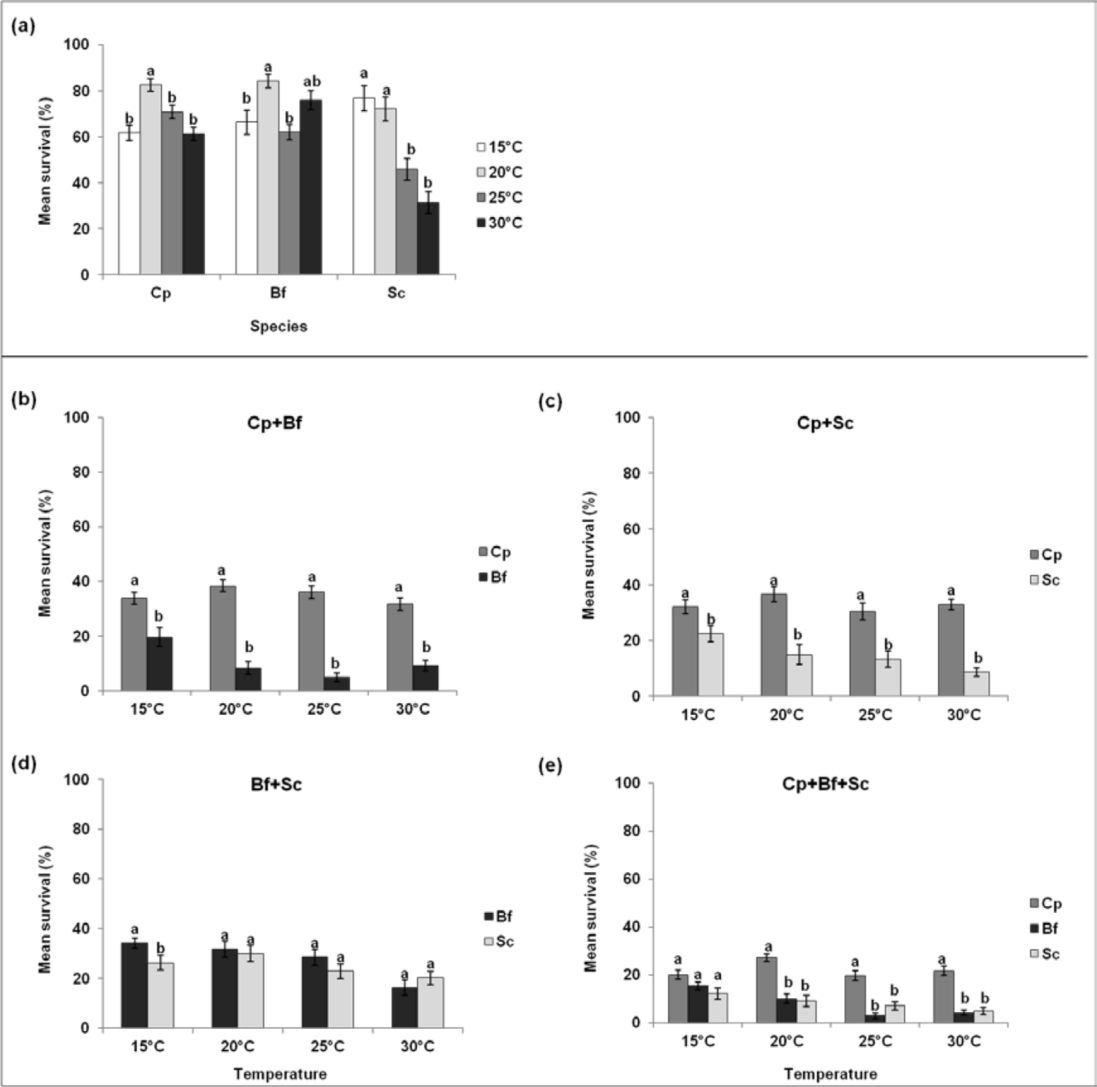
**Comparison of survival of *Chilo partellus* (Cp), *Busseola fusca* (Bf) and *Sesamia calamistis* (Sc) larvae as single-species (a) and between borer species in multi-species communities at different constant temperatures (b-e)**. Means (± SE) with different letters are significantly different at 5% level. GLM (binomial).

**Fig 5:**
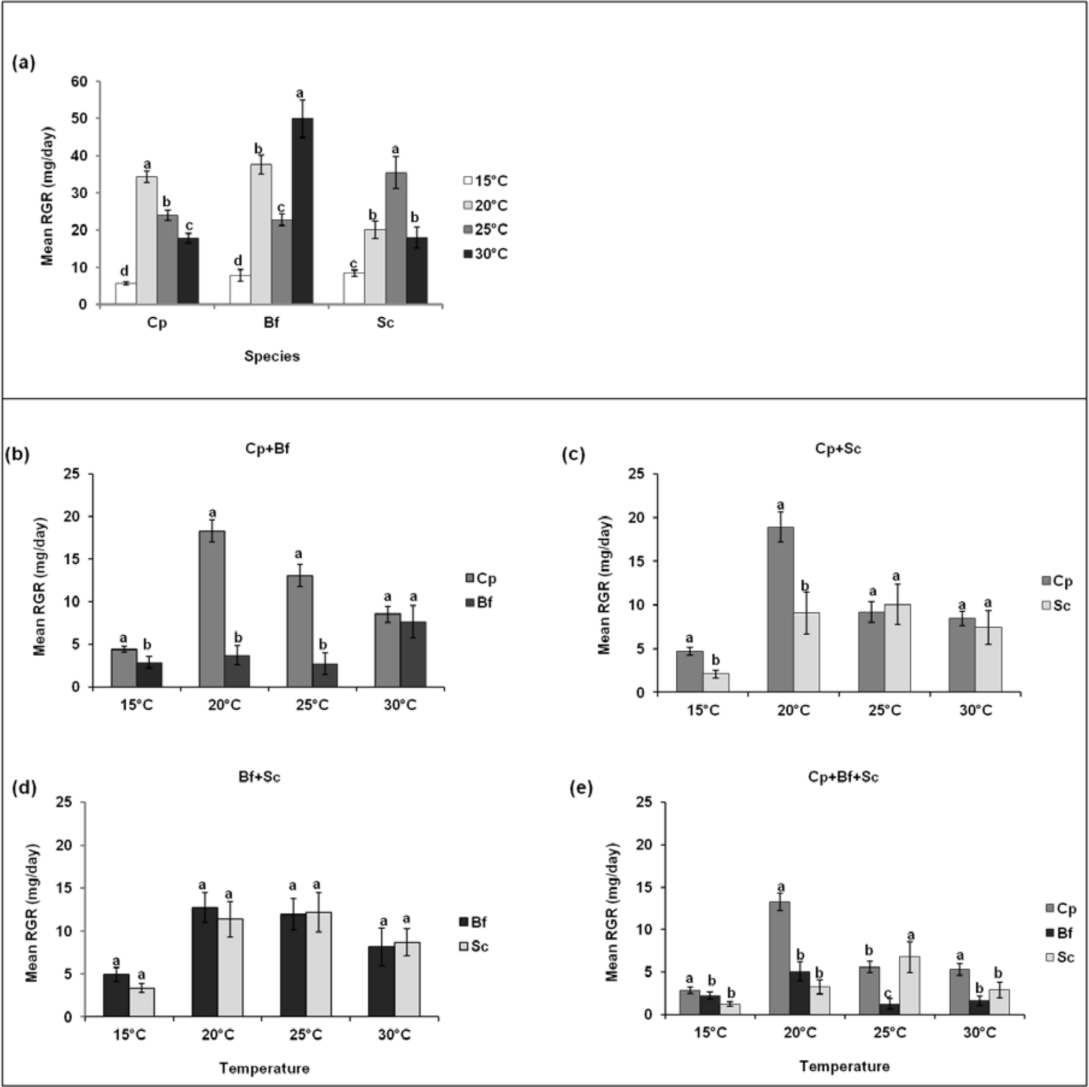
**Comparison of the relative growth rates (RGR) of *Chilo partellus* (Cp), *Busseola fusca* (Bf) and *Sesamia calamistis* (Sc) larvae as single-species (a) and between borer species in multi-species communities at different constant temperatures (b-e)**. Means (± SE) with different letters are significantly different at 5% level according to the Student-Newman-Keuls test.

For each species, larval survival in the single-species treatments varied significantly between temperatures (Fig 4A). For *B*. *fusca*, it was highest at 20°C and lowest at 25°C. For *C*. *partellus*, it was highest at 20°C and similar among the other temperatures, while for *S*. *calamistis* it was higher at 15°C and 20°C than 25°C and 30°C (Table 1). RGR of each species was lowest at 15°C (Fig 5A). For *S*. *calamistis*, it was similar at 20°C and 30°C and highest at 25°C, while for *B*. *fusca*, it was highest at 30°C. For *C*. *partellus*, the highest RGR was recorded at 20°C, whereafter it decreased with increasing temperature (Fig 5A, Table 2).

**Table 1:** Results of GLM analysis comparing larval survival of each single-species at different constant temperatures.

	*Chilo partellus*		*Busseola fusca*		*Sesamia calamistis*	
Temperature	O.R. (95% CI)	P-value	O.R. (95% CI)	P-value	O.R. (95% CI)	P-value
**15°C**	1		1		1	
**20°C**	2.9 (1.9–4.6)	<0.001	2.7 (1.5–5.0)	0.002	0.8 (0.4–1.6)	0.51
**25°C**	1.5 (1.0–2.2)	0.04	0.8 (0.5–1.4)	0.49	0.3 (0.1–0.5)	<0.001
**30°C**	1.0 (0.7–1.4)	0.93	1.6 (0.9–2.8)	0.1	0.1 (0.1–0.3)	<0.001

O.R. = Odd Ratios.

**Table 2:** Results of ANOVA comparing relative growth rates of each single-species at different constant temperatures.

Species	F	P-value
***Chilo partellus***	94.1	<0.001
***Busseola fusca***	36.5	<0.001
***Sesamia calamistis***	15.0	<0.001

#### b) Comparison of survival and RGR of *B*. *fusca*, *C*. *partellus* and *S*. *calamistis* in multi-species communities under different constant temperatures

Survival was higher for *C*. *partellus* than its companion species at all temperatures (Fig 4B and 4C, e and Table 3). In pairings with *B*. *fusca* and *S*. *calamistis*, survival was similar between the two species at all temperatures except at 15°C (Fig 4D). In pairings with *C*. *partellus*, the crambid had significantly higher RGRs than *B*. *fusca* at all temperatures except at 30°C (Fig 5B) and also higher than *S*. *calamistis* at 15°C and 20°C (Fig 5C). In pairings involving both noctuids, RGRs did not vary significantly between the two species regardless of the temperature (Fig 5D), except for the 3-species pairing at 25°C where *S*. *calamistis* had a higher RGR than *B*. *fusca* (Fig 5E, Table 4).

**Table 3:** Results of GLM analysis comparing larval survival between borer species in multi-species communities at different constant temperatures.

	Cp+Bf		Cp+Sc		Bf+Sc		Cp+Bf+Sc (Cp *vs* Bf)		Cp+Bf+Sc (Bf *vs* Sc)	
Temperature	O.R. (95% CI)	P-value	O.R. (95% CI)	P-value	O.R. (95% CI)	P-value	O.R. (95% CI)	P-value	O.R. (95% CI)	P-value
**15°C**	3.2 (1.6–6.6)	0.002	0.5 (0.2–0.8)	0.02	0.5 (0.3–0.9)	0.03	1.7 (0.9–3.6)	0.13	0.7 (0.3–1.3)	0.3
**20°C**	16.4 (7.7–37.2)	<0.001	0.2 (0.1–0.4)	<0.001	0.9 (0.4–1.9)	0.71	10.1 (4.2–26)	<0.001	0.9 (0.4–2)	0.78
**25°C**	26.5 (11.8–65.9)	<0.001	0.2 (0.1–0.5)	<0.001	0.6 (0.3–1.3)	0.21	14.9 (6–43.1)	<0.001	2.8 (1.1–8.5)	0.05
**30°C**	7.7 (4.1–14.8)	<0.001	0.1 (0.1–0.2)	<0.001	1.4 (0.7–2.9)	0.35	13 (5.8–31.7)	<0.001	1.2 (0.5–3.2)	0.66

O.R. = Odd Ratios, *Chilo partellus* (Cp), *Busseola fusca* (Bf) and *Sesamia calamistis* (Sc).

**Table 4:** Results of ANOVA comparing relative growth rates between borer species in multi-species communities at different constant temperatures.

	Cp+Bf		Cp+Sc		Bf+Sc		Cp+Bf+Sc	
Temperature	F	P-value	F	P-value	F	P-value	F	P-value
**15°C**	4.1	0.04	17.3	<0.001	2.4	0.13	5.1	0.009
**20°C**	70.2	<0.001	11.0	0.002	0.3	0.61	27.4	<0.001
**25°C**	31.6	<0.001	0.1	0.74	0.01	0.94	6.0	0.004
**30°C**	0.2	0.67	0.3	0.62	0.04	0.84	6.4	0.003

*Chilo partellus* (Cp), *Busseola fusca* (Bf) and *Sesamia calamistis* (Sc).

#### c) Comparison of survival and relative growth rates between single and multi-species communities of *B*. *fusca*, *C*. *partellus* and *S*. *calamistis* at different constant temperatures

**Fig 6:**
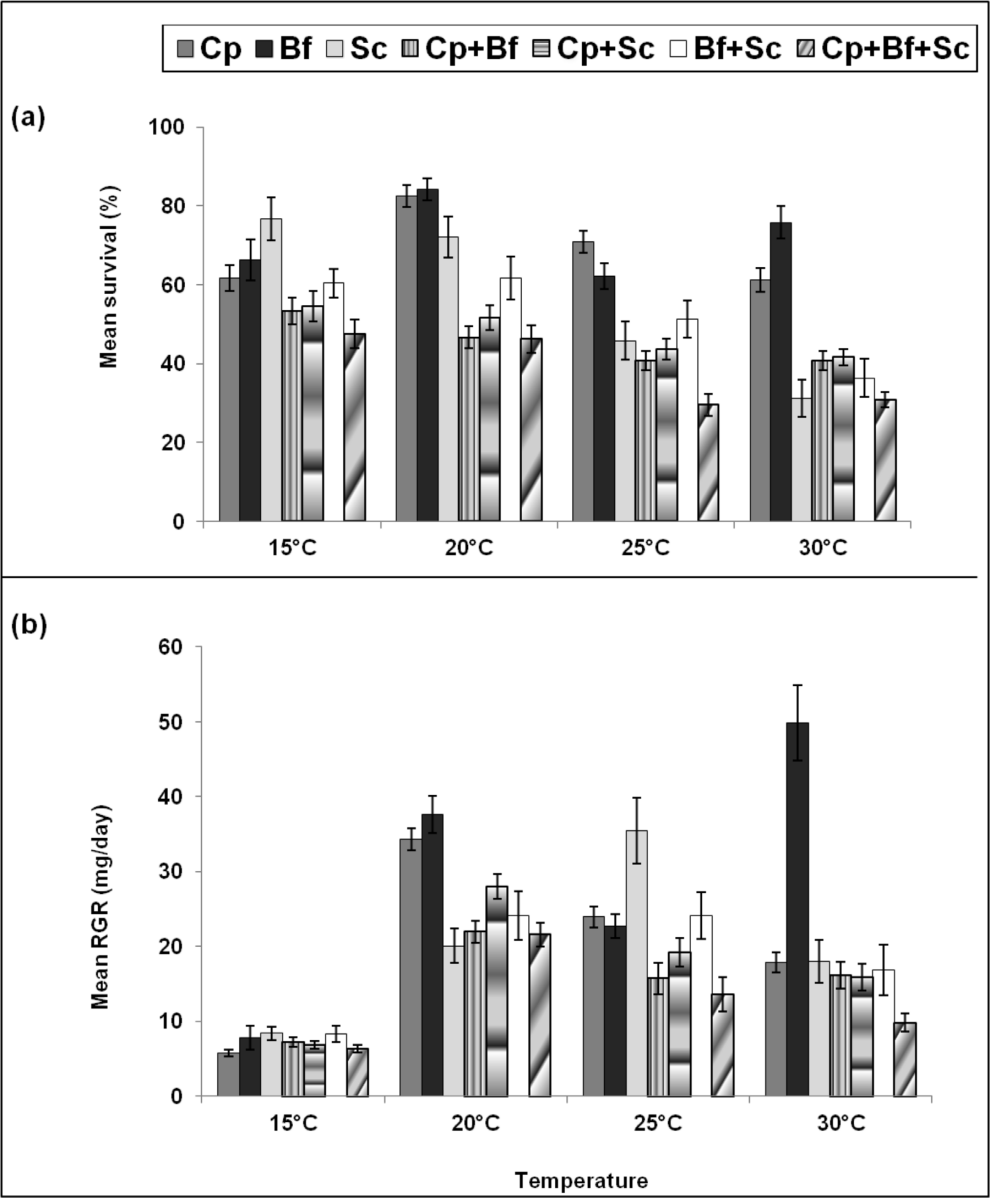
**Comparative survival (a) and RGR (b) between single-species and multi-species communities of *Chilo partellus* (Cp), *Busseola fusca* (Bf) and *Sesamia calamistis* (Sc) under different constant temperatures**. Statistical comparisons were only made between single- and the corresponding multi-species pairings (see Tables 5 and 6).

When significant, survival and RGR of single species communities were higher than total survival and RGR of the corresponding multi-species communities. Between 20–30°C, survival of *B*. *fusca*, and *C*. *partellus* singly tended to be higher than total survival of the corresponding multi-species communities. For *S*. *calamistis*, it was higher than that of multi-species communities at 15° and 20°C, and to a lesser extent at 25° and 30°C (Fig 6A, Table 5). Likewise between 20° and 30°C, RGRs of single-species communities of *C*. *partellus*, *B*. *fusca*, and to a lesser extent of *S*. *calamistis* tended to be higher than total RGRs of the corresponding multi-species communities (Fig 6B, Table 6).

**Table 5:** Results of GLM analysis comparing survival between single-species and multi-species communities under different constant temperatures.

	Temperature							
	15°C		20°C		25°C		30°C	
Treatment comparisons	O.R. (95% CI)	P-value	O.R. (95% CI)	P-value	O.R. (95% CI)	P-value	O.R. (95% CI)	P-value
Cp *vs* Cp+Bf	1.4 (1.0–2.1)	0.08	5.4 (3.6–8.2)	<0.001	3.5 (2.4–5.2)	<0.001	2.3 (1.6–3.3)	<0.001
Cp *vs* Cp+Sc	0.7 (0.5–1.1)	0.17	0.2 (0.1–0.3)	<0.001	0.3 (0.2–0.5)	<0.001	0.5 (0.3–0.6)	<0.001
Cp *vs* Cp+Bf+Sc	1.8 (1.2–2.7)	0.007	5.5 (3.5–8.5)	<0.001	5.8 (3.9–8.6)	<0.001	3.5 (2.4–5.2)	<0.001
Bf *vs* Cp+Bf	1.7 (1.0–2.9)	0.05	6.1 (3.9–9.6)	<0.001	2.4 (1.6–3.5)	<0.001	4.5 (2.9–7.3)	<0.001
Bf *vs* Bf+Sc	0.8 (0.4–1.3)	0.37	0.3 (0.2–0.6)	<0.001	0.6 (0.4–1.0)	0.06	0.2 (0.1–0.3)	<0.001
Bf *vs* Cp+Bf+Sc	2.2 (1.3–3.7)	0.007	6.2 (3.8–10.2)	<0.001	3.9 (2.7–5.7)	<0.001	7.0 (4.4–11.3)	<0.001
Sc *vs* Bf+Sc	2.2 (1.1–4.1)	0.02	1.6 (0.8–3.2)	0.17	0.8 (0.5–1.4)	0.4	0.8 (0.4–1.4)	0.4
Sc *vs* Cp+Sc	2.7 (1.5–5.3)	0.003	2.4 (1.4–4.2)	0.002	1.1 (0.7–1.7)	0.71	0.6 (0.4–1.0)	0.05
Sc *v*s Cp+Bf+Sc	3.6 (1.9–6.9)	<0.001	3.0 (1.7–5.3)	<0.001	2.0 (1.3–3.2)	0.005	1.0 (0.6–1.6)	0.9

O.R. = Odd Ratios, *Chilo partellus* (Cp), *Busseola fusca* (Bf) and *Sesamia calamistis* (Sc).

**Table 6:** Results of ANOVA comparing the relative growth rates between single-species and multi-species communities under different constant temperatures.

	Temperature											
		15°C			20°C			25°C			30°C	
	Treatment comparisons	F	P-value		F	P-value		F	P-value		F	P-value
	Cp *vs* Cp+Bf	3.9	0.05		35.2	<0.001		10.8	0.002		0.6	0.43
	Cp *vs* Cp+Sc	2.7	0.11		8.0	0.007		3.9	0.05		0.8	0.37
	Cp *vs* Cp+Bf+Sc	0.8	0.39		35.3	<0.001		14.9	<0.001		20.7	<0.001
	Bf *vs* Cp+Bf	0.1	0.74		30.1	<0.001		7.3	0.01		39.9	<0.001
	Bf *vs* Bf+Sc	0.1	0.81		11.1	0.001		0.2	0.69		29.1	<0.001
	Bf *vs* Cp+Bf+Sc	0.8	0.39		30.6	<0.001		10.9	0.002		59.8	<0.001
	Sc *vs* Bf+Sc	0.0	0.94		1.0	0.31		4.3	0.04		0.1	0.80
	Sc *vs* Cp+Sc	2.3	0.14		7.9	0.007		11.5	0.001		0.4	0.54
	Sc *vs* Cp+Bf+Sc	3.6	0.06		0.3	0.60		19.6	<0.001		6.9	0.01

*Chilo partellus* (Cp), *Busseola fusca* (Bf) and *Sesamia calamistis* (Sc).

## Discussion

For each species, survival was reduced when the larval density doubled. Also, when reared together with one or several species, survival and RGR of each species tended to decrease compared to the single-species treatment. Thus, the intra- and interspecific interactions between the stemborer species tested in this study indicated competitive resource utilization. An inverse effect of density on species fitness and their interactions is an established ecological fact [47–49] and a typical characteristic of spatially restricted insects such as Lepidoptera living inside a stem and exploiting the same resources [11, 19, 20, 50]. Cereal stemborers in East Africa constitute an extreme case of interactions for resource utilisation. Their larvae have developed a close association with their host plants [51] as they coexist with a “restricted” resource, available over a short period of time (2 to 3 months), with the most nutritious stage between 2 to 8 weeks and with unreliable availability of suitable hosts because drought spells commonly occur in the region. All these characteritics make cereal stemborers a good model for testing the competition theory [11, 20, 52].

Interspecific competition was more pronounced than intraspecific competition, especially when *C*. *partellus* was involved, with the outcomes skewed asymmetrically towards the crambid. This indicates a higher fitness of the crambid compared to the two noctuids. This confirms the asymmetry of interspecific competition outcomes in phytophagous insects [11, 20, 53]. Thus in favourable regions where the crambid co-occurs with either one or both noctuids in infesting maize or any cereal crop, the crambid will likely dominate over the other two species.

The superior competitive abilities of *C*. *partellus* over other species have been reported from other field and laboratory studies. In South Africa, *C*. *partellus* was reported to be superior to *B*. *fusca* in colonizing ratoon sorghum and its population build-up occurred faster [41]. Furthermore, a comparison of life traits using five grasses showed that the invasive *C*. *partellus* laid more viable eggs, its larvae consumed more food and had a higher survival and shorter developmental rate than the native *C*. *orichalcociliellus* [54]. Various studies have described the superior competitive abilities of invasive over native species. For instance, the superior competitive abilities of several invasive species as a key factor for their successful establishment has been well documented [55]. The proficiency in both interference and exploitative competition was also reported to confer a superior ability on the invasive Argentine ant, *Linepithema humile* Mayr (Hymenoptera: Formicidae) over native species [56].

The competition-relatedness hypothesis states that closely related species will compete stronger than distantly related species [11, 57]. In contrast, in the present study, competition was stronger between distant-related species (noctuids and crambid) than between the two noctuids belonging to the same sub-tribe. Similarly, results from a meta-analysis concluded that distant-relatedness rather than phylogenetic similarity determined the strength of competition in insects [20]. The present study thus confirms others that disputes the competition-relatedness hypothesis [58–60].

This study demonstrated that temperature is an important factor influencing the interactions between the noctuids and the crambid. Thereby, the competitive abilities of each of the species depended on its temperature tolerance limits for development. While high temperatures favoured *C*. *partellus*, the two noctuids had highest survival rates under lower temperatures. Likewise, as shown previously [25, 26], the development rate of the three species increased with temperature but this was more pronounced for *C*. *partellus* and *S*. *calamistis* than *B*. *fusca*. In the field, while *C*. *partellus* and *B*. *fusca* dominate within a limited thermal tolerance at the high and low temperature extremes, respectively, *S*. *calamistis* has a wider thermal tolerance by co-occuring with the two species along these temperature gradients [7, 9, 10].

The role of temperature in influencing varied competitive abilities of interacting species has been reported from three *Drosophila* species [61], between small and large seed beetle species *Stator limbatus* [34], the invasive fruit fly *Bactrocera invadens* Drew, Tsuruta & White over the indigenous fruit fly, *Ceratitis cosyra* (Walker) (Diptera: Tephritidae) [62] and two invasive leaf miner flies *Liriomyza sativae* Blanchard and *L*. *trifolii* (Burgess) (Diptera: Agromyzidae) [63]. In these studies, the competitive abilities of one of the competing species was enhanced by either low or high temperatures. Similar trends of temperature influence have been reported from competition studies involving plants [64], fish [65–67] and bacterivorous ciliates [68].

Several studies have been conducted to assess the potential impacts of climate change on various life history parameters of insects such as their population dynamics, survival and mass and their distribution [28, 69–73]. However, few studies exist on the effects of temperature increase on the interactions of species using the same resources [39]. Results of the present study suggest that a future increase in temperature would confer a greater competitive ability on *C*. *partellus* than the two noctuid species. Similarly, temperature-dependent models predicted that *C*. *partellus* will expand into the highlands where *B*. *fusca* presently dominates [26]. With its better competitive abilities, *C*. *partellus* is likely to outcompete the two noctuids in the highlands and become the dominant species. In fact, *C*. *partellus* has already been recorded from highlands in Kenya and cooler regions of South Africa, and in some cases it has become the dominant species [4, 7, 9, 10, 41, 42]. This is also likely to increase the level of damage to cereal crops in these high elevation regions, given that *C*. *partellus* causes more injury than *B*. *fusca* on maize in some regions [5, 7]. Similar observations of a potential increase in crop damages by other insect pests, caused by temperature increase due to climate change, have been reported [28, 74, 75].

As shown by higher survival and RGRs for *C*. *partellus* and *S*. *calamistis*, surrogate stems were a good alternative to maize plants. Although the RGRs were not significantly higher in surrogates stems compared to maize plants for *B*. *fusca*, the use of surrogate stems for this species was also a good alternative to maize plants since its survival increased almost by twofold in surrogtae stems compared to maize plants. In general, insects tend to perform better on artificial than on natural diets since artificial diets possess optimum levels of nutrients and vitamins [76]. However in nature, early instars of *C*. *partellus* and *B*. *fusca* migrate by “ballooning off” the plant [77, 78], which is not possible when surrogate stems are used. Thus, whether the higher survival on surrogate stems were due to lower mortality or reduced migration could not be determined with the present experimental set-up. Still, surrogate stems are more stable than maize stems or potted plants because they do not deteriorate that easily and are thus ideal for such studies. Similarly, higher survival of *S*. *calamistis* reared on artificial diet than on maize stem cuttings was reported by other authors [79].

This study highlights the knowledge gap in our understanding of temperature effects on biodiversity, especially interactions between species utilizing the same resources. Besides temperature, rainfall is another abiotic factor which could influence interactions within stemborer communities [80–84]. In addition, biotic factors such as density dependence [48], level of multi-species infestations in the field and oviposition-site selection of the female adults [85–87] could also influence stemborer species interactions. Further studies which elucidate the influences of these factors will enable a better understanding of the impact of stemborer interactions on cereal crop damage, especially under future climate scenarios and contribute to the development of possible mitigation and adaption strategies.
